# Supplementary material for: Mesenchymal Stem Cell Microvesicles from Adipose Tissue: Unraveling Their Impact on Primary Ovarian Cancer Cells and Their Therapeutic Opportunities
Source: Int J Mol Sci. 2023 Nov 1;24(21):15862. doi: 10.3390/ijms242115862 (PMC10647545; doi:10.3390/ijms242115862)
Supplement: Supplementary file 1 [file ijms-24-15862-s001.zip › Figure S1.pdf]

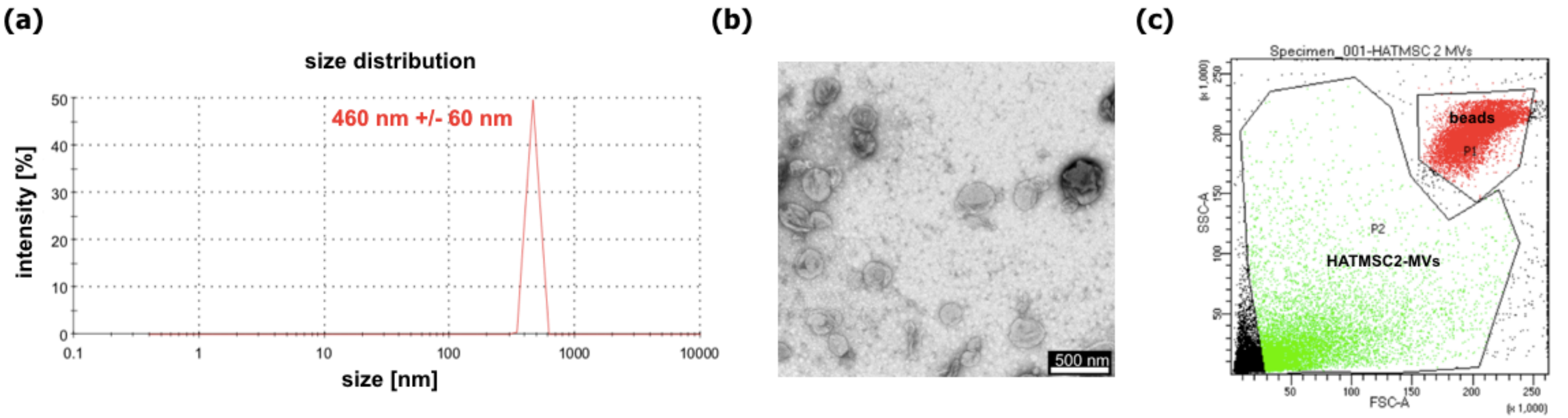

Figure S1. HATMSC2-MVs characteristics. **(a)** Representative histogram from dynamic light scattering (DLS) analysis. **(b)** Representative images from transmission electron microscopy (TEM) imaging. (c) Dot plot from flow cytometry analysis illustrating the forward scatter (FSC) versus side scatter (SSC). P1 represents counting beads, P2 represents HATMSC2-MVs. Bar represents 500 nm HATMSC2-MVs—microvesicles derived from immortalized human mesenchymal stem cells of adipose tissue origin.
