# Supplementary material for: Mesenchymal Stem Cell Microvesicles from Adipose Tissue: Unraveling Their Impact on Primary Ovarian Cancer Cells and Their Therapeutic Opportunities
Source: Int J Mol Sci. 2023 Nov 1;24(21):15862. doi: 10.3390/ijms242115862 (PMC10647545; doi:10.3390/ijms242115862)
Supplement: Supplementary file 1 [file ijms-24-15862-s001.zip › Table S1.pdf]

Table S1. List of primary antibodies

Antigen	Host	Class of antibody	Clone	Company	Dilution	Incubation
Human PDGFRa	Mouse	Monoclonal	C-9	Santa Cruz	1:100	1h, Room Temperature (RT)
Human CD44	Rabbit	Polyclonal		Thermo Fisher Scientific	1:100	1h, RT
Human Fibroblast activation protein (FAP)	Mouse	Monoclonal	F11-24	Thermo Fisher Scientific	1:100	Overnight (O/N), 4^0C
Human Snail1	Rabbit	Polyclonal		Bio Site	1:100	O/N, 4^0C
Human Oct4	Rabbit	Polyclonal		Thermo Fisher Scientific	1:100	O/N, 4^0C
Human Sox2	Mouse	Monoclonal	20 G5	Thermo Fisher Scientific	1:100	O/N, 4^0C
Human Nanog	Rabbit	Polyclonal		Thermo Fisher Scientific	1:100	O/N, 4^0C
Human ALDH1	Rabbit	Polyclonal		Thermo Fisher Scientific	1:200	1h, RT
Human vimentin	Mouse	Monoclonal	V9	Thermo Fisher Scientific	1:100	1h, RT
Human c-kit (CD177)	Mouse	Monoclonal	K45	Thermo Fisher Scientific	1:50	O/N, 4^0C
