# Supplementary material for: Mesenchymal Stem Cell Microvesicles from Adipose Tissue: Unraveling Their Impact on Primary Ovarian Cancer Cells and Their Therapeutic Opportunities
Source: Int J Mol Sci. 2023 Nov 1;24(21):15862. doi: 10.3390/ijms242115862 (PMC10647545; doi:10.3390/ijms242115862)
Supplement: Supplementary file 1 [file ijms-24-15862-s001.zip › Table S2.pdf]

Table S2. List of secondary antibodies

Antigen	Host	Class of antibody	Flouorophore	Company	Dilution	Incubation
F-actin		Phalloidin	Alexa Fluor 488™	Thermo Fisher Scientific	1:400	45min, RT
Mouse IgG	Goat	Polyclonal	Alexa Fluor 488™	Thermo Fisher Scientific	1:700	45min, RT
Mouse IgG	Goat	Polyclonal	Alexa Fluor 647™	Thermo Fisher Scientific	1:700	45min, RT
Rabbit IgG	Goat	Polyclonal	Alexa Fluor 647™	Thermo Fisher Scientific	1:700	45min, RT
